# The Genetic Basis of Fuchs Endothelial Corneal Dystrophy

**Published:** 2010-10

**Authors:** Sepehr Feizi

**Affiliations:** Ophthalmic Research Center, Shahid Beheshti University of Medical Sciences, Tehran, Iran

Fuchs endothelial corneal dystrophy (FECD) is characterized by pleomorphic, attenuated, dysfunctional, and degenerated corneal endothelium together with progressive formation of corneal guttae. The condition may show familial clustering but is usually sporadic and predominantly affects women. Family based studies have mapped late onset FECD susceptibility to 13ptel-13q12.13 and 18q21.2-q21.32. Genome-wide linkage analysis has identified potential linkage regions on chromosomes 1, 7, 15, 17, and X. Recently FECD has been linked to a novel locus on 5q33.1-q35.2. Mutations in the *COL8A2* gene located on 1p34.3 have also been described in patients with FECD.

Heterozygous mutations in the *SLC4A11* gene are known to be associated with late-onset FECD. The *SLC4A11* gene, which codes for sodium bicarbonate transporter-like protein 11, has been previously associated with autosomal recessive congenital hereditary endothelial dystrophy (CHED2) which is also classified as a primary defect of the corneal endothelium.

Recently, Hemadevi et al screened for mutations in *COL8A2* and *SLC4A11* genes to determine their contribution to FECD in an Indian population. They did not identify any pathogenic mutations in *COL8A2* in association with FECD, although previous studies on other ethnic groups had reported such mutations. In the *SLC4A11* screening they identified 2 novel and 3 previously reported silent variants which had no significant association with FECD, however the investigators identified no pathogenic variants. Based on these observations and previous studies, they concluded that locus heterogeneity exists for FECD, whereby mutations in several genes on different chromosomes may lead to a common disease phenotype.

## References

[1] Hemadevi B, Srinivasan M, Arunkumar J, Prajna NV, Sundaresan P (2010). Genetic analysis of patients with Fuchs endothelial corneal dystrophy in India. BMC Ophthalmol.

